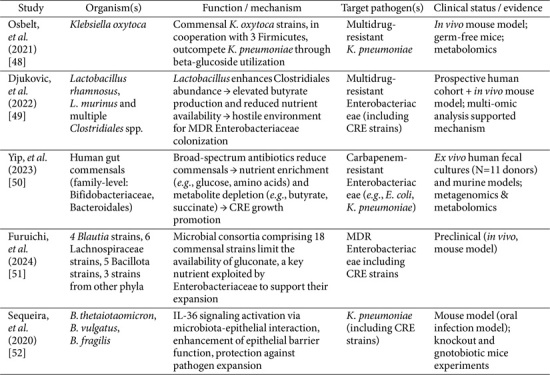# Gut Microbiome-Based Strategies for the Control of Carbapenem-Resistant Enterobacteriaceae

**DOI:** 10.4014/jmb.2406.06017

**Published:** 2025-08-07

**Authors:** Imchang Lee, Bong-Soo Kim, Ki Tae Suk, Seung Soon Lee

**Affiliations:** 1 Division of Infectious Diseases, Department of Internal Medicine, Hallym University Chuncheon Sacred Heart Hospital, Hallym University College of Medicine, Chuncheon, Republic of Korea; 2 Department of Nutritional Science and Food Management, Ewha Womans University, Seoul, Republic of Korea; 3 Division of Gastroenterology and Hepatology, Department of Internal Medicine, Hallym University Chuncheon Sacred Heart Hospital, Hallym University College of Medicine, Chuncheon, Republic of Korea; 4 Institute for Liver and Digestive Diseases, Hallym University, Chuncheon, Republic of Korea

**Keywords:** Carbapenem-resistant enterobacteriaceae, gastrointestinal microbiome, colonization resistance, microbiome disruption index, fecal microbiota transplantation, symbiotic microbial consortia

## Abstract

Carbapenem-resistant Enterobacteriaceae (CRE) represent a critical antimicrobial resistance threat due to their resistance to last-resort antibiotics and high transmission potential. While conventional strategies—such as infection control, antimicrobial stewardship, and novel antibiotic development—remain essential, growing attention has shifted toward the gut microbiome, which plays a central role in mediating colonization resistance against CRE. Disruption of the intestinal microbiota—primarily driven by antibiotic exposure and further exacerbated by non-antibiotic drugs such as proton pump inhibitors—reduces microbial diversity and impairs functional integrity, facilitating CRE acquisition, prolonged carriage, and horizontal transmission. In response, microbiome-based strategies—including microbiome disruption indices (MDIs), fecal microbiota transplantation (FMT), and rationally designed symbiotic microbial consortia—are being explored as novel approaches for CRE prevention and decolonization. Mechanistic studies have shown that colonization resistance is mediated by both direct mechanisms (*e.g.*, nutrient competition, short-chain fatty acid production) and indirect mechanisms (*e.g.*, immune modulation via IL-36 signaling). Advances in metagenomics, metabolomics, and culturomics have enabled high-resolution profiling of gut microbial communities and their functional roles. Emerging preclinical and clinical evidence supports the potential of microbiome-informed interventions to predict infection risk, enhance antimicrobial stewardship, and guide the development of next-generation probiotics targeting CRE. Longitudinal studies continue to evaluate the efficacy of FMT and synthetic microbial consortia in eradicating intestinal CRE colonization. Collectively, these insights underscore the promise of gut microbiome science as a complementary and innovative strategy for CRE control in the post-antibiotic era.

## Introduction

Carbapenem-resistant Enterobacteriaceae (CRE), including *Klebsiella pneumoniae*, *Escherichia coli*, and *Enterobacter* spp., represent a serious global health threat due to their resistance to nearly all β-lactam antibiotics, limited treatment options, and high transmission potential in healthcare settings [1, 2]. The U.S. Centers for Disease Control and Prevention (CDC) classifies CRE as an “urgent threat,” while the World Health Organization (WHO) designates it as a “critical priority pathogen” [3, 4]. In South Korea, following the implementation of mandatory national surveillance in 2016, the number of reported CRE cases has increased more than fourfold—from approximately 800 cases per month in 2016 to over 3,500 per month in 2024 [5].

CRE commonly colonize the gastrointestinal tract without causing immediate symptoms; however, this asymptomatic colonization frequently precedes invasive infections such as bacteremia, pneumonia, or urinary tract infections [6]. The gut serves as a persistent reservoir and plays a central role in CRE transmission within healthcare settings [7]. Longitudinal studies have shown that colonization by CRE, particularly *K. pneumoniae* carbapenemase (KPC)-producing strains, can persist for months or even years in hospitalized or frequently readmitted patients [8, 9].

Disruption of the gut microbiota—primarily driven by antibiotic exposure and, in some cases, further exacerbated by co-administration of non-antibiotic agents such as proton pump inhibitors (PPIs)—can reduce microbial diversity and impair colonization resistance [10, 11]. This dysbiosis facilitates CRE acquisition, expansion, and horizontal gene transfer of resistance elements within the intestinal ecosystem [10, 12-15]. While partial recovery of the microbiota may occur after short-term antibiotic exposure in healthy individuals, prolonged or repeated administration of broad-spectrum antibiotics often results in sustained dysbiosis and heightened susceptibility to colonization by resistant organisms [16, 17].

Given the limitations of conventional infection control and passive decolonization approach, increasing attention has turned to microbiome-based strategies aimed at preserving or restoring colonization resistance [18]. In this review, we examine microbiome-informed approaches for CRE control, structured around the CDC’s four core actions to combat antibiotic resistance: preventing infections, tracking resistance, improving antibiotic use, and developing new treatments [3]. Particular emphasis is placed on microbiome disruption indices (MDIs), fecal microbiota transplantation (FMT), and defined symbiotic microbial consortia as promising adjunctive strategies. Unlike previous reviews that have primarily focused either on antimicrobial stewardship or on the therapeutic use of fecal microbiota transplantation in isolation, this review provides an integrative framework that connects mechanistic insights into colonization resistance with the emerging concept of MDIs. These insights highlight the potential of MDIs to inform risk prediction for infection prevention, guide stewardship interventions targeting both antibiotics and PPIs, and support the development of next-generation microbiome-based therapeutics specifically targeting CRE. By synthesizing recent clinical and preclinical evidence, this review aims to bridge critical knowledge gaps between ecological vulnerability assessment, stewardship strategies, and the rational design of defined microbial consortia for CRE decolonization.

## Gut Microbiome-Based Strategies for Preventing CRE Infections and Controlling Their Spread

Preventing infections caused by CRE requires proactive strategies that address not only pathogen exposure but also host susceptibility—particularly the ecological vulnerability of the gut microbiome. CRE colonization of the gastrointestinal tract often precedes systemic infections and plays a key role in healthcare-associated transmission. Microbiota-disrupting exposures—such as antibiotics and PPIs—compromise colonization resistance, facilitating CRE persistence and overgrowth [6, 7, 10-12].

**Fig. 1:**
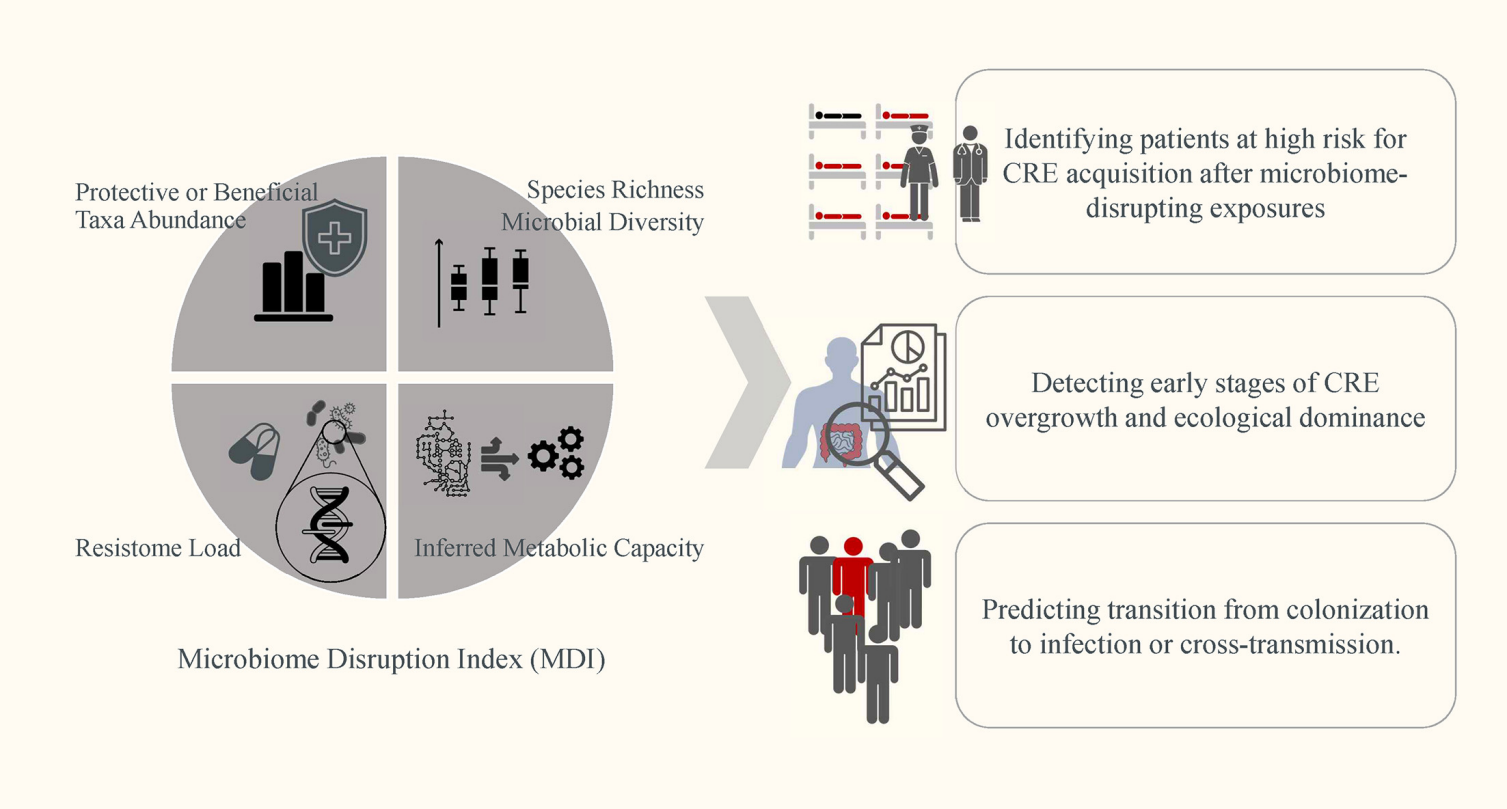
Key components and predictive applications of the Microbiome Disruption Index (MDI) for CRE risk stratification. MDI quantifies gut microbiome disruption using four components: (i) microbial richness and diversity, (ii) abundance of protective taxa, (iii) resistome burden, and (iv) inferred metabolic capacity. These metrics help stratify carbapenem-resistant Enterobacteriaceae (CRE) risk after microbiome-disrupting exposures, detect early overgrowth, and predict infection or transmission.

In this context, the U.S. CDC has introduced the concept of MDIs as a tool to identify patients at elevated risk of acquiring antimicrobial-resistant organisms, including CRE [18]. MDIs integrate various ecological and functional parameters—such as species richness, microbial diversity, the abundance of beneficial or protective taxa, resistome load, and inferred metabolic capacity—to quantify the degree of microbiome disruption and predict susceptibility to colonization, overgrowth, and infection or cross-transmission. In CRE-specific applications, MDIs may support infection prevention efforts in several ways (Fig. 1): (i) Identifying patients at high risk for CRE acquisition after microbiome-disrupting exposures (*e.g.*, antibiotics, PPIs) [19-21]; (ii) Detecting early stages of CRE overgrowth and ecological dominance, which may precede systemic infection; (iii) Predicting transition from colonization to CRE-related infection or nosocomial transmission, enabling timely intervention [21, 22]. Fig. 1 summarizes how these MDI components work synergistically to capture the ecological vulnerability of the gut, thereby enabling real-time, microbiome-informed risk stratification for CRE in clinical settings.

A metagenomic study of 98 intensive care unit (ICU) patients demonstrated that individuals colonized with CRE exhibited significantly lower microbial diversity and taxonomic alterations compared to non-colonized patients [21]. At the phylum level, CRE carriers showed decreased abundances in Bacteroidota (formerly Bacteroidetes) and Actinomycetota (formerly Actinobacteria), and an increased abundance of Pseudomonadota (formerly Proteobacteria). Notably, species-level analyses revealed significant abundance of *K. pneumoniae* and *Enterococcus faecium*—organisms commonly associated with hospital-acquired infections and horizontal gene transfer of resistance genes. These findings reinforce the role of gut dysbiosis in promoting CRE colonization and suggest that MDIs may serve as useful biomarkers for stratifying CRE infection risk. In addition to this study, several other key investigations have provided valuable insights into MDI-based approaches for assessing CRE risk. Korach-Rechtman *et al*. [19] reported intestinal dysbiosis in CRE carriers, although they did not identify specific taxonomic drivers. In contrast, Seekatz *et al*. [20] emphasized the predictive utility of both microbiome composition and clinical variables present at the time of hospital admission. Shimasaki *et al*. [22] found that an increased relative abundance of KPC-producing *K. pneumoniae* within the gut microbiota was associated with subsequent bloodstream infection risk, highlighting the prognostic potential of microbiome-based indices. These studies, summarized in Table 1, collectively illustrate both the commonalities and methodological distinctions that contribute to the development and validation of MDIs across diverse clinical settings.

Despite their potential, the clinical integration of MDIs remains limited by technical and logistical challenges [23-25]. These include the high cost of metagenomic sequencing, lack of standardized threshold values for defining microbiome disruption, and insufficient longitudinal datasets linking MDIs to clinical endpoints such as infection or transmission. As sequencing technologies become more cost-effective and hospital-based microbiome surveillance infrastructures mature, precision-guided infection control programs incorporating MDIs are likely to become feasible components of standard infection prevention strategies. For example, MDIs could be used to stratify high-risk patients in intensive care units for targeted surveillance, prioritize candidates for microbiome-restorative therapies such as FMT, or guide selective antibiotic use in antimicrobial stewardship programs. In the future, integration of real-time MDIs into electronic health record (EHR) systems may enable dynamic risk profiling and automated infection control alerts tailored to a patient’s microbiome status.

## Gut Microbiome-Based Strategies for Antimicrobial and PPI Stewardship in CRE Control

Antibiotic exposure is a well-established risk factor for intestinal CRE colonization [26]. By disrupting the gut microbiota, antibiotics reduce microbial diversity and compromise colonization resistance, thereby creating ecological conditions favorable for CRE acquisition and persistence [10, 14]. MDIs were developed not only for infection control but also for use in antimicrobial stewardship, particularly in preventing CRE colonization, particularly in preventing CRE colonization. Unlike conventional biomarkers such as procalcitonin, which guide antibiotic discontinuation based on clinical resolution of infection, MDIs assess the ecological vulnerability of the gut microbiota and estimate the risk of colonization by antimicrobial-resistant organisms under ongoing antibiotic pressure [18, 27]. Incorporating MDIs into antimicrobial stewardship frameworks facilitates the identification and risk stratification of patients likely to acquire or maintain CRE due to microbiome disruption. This approach promotes the preservation of colonization resistance and reduces selective pressure, thereby enabling more targeted and ecologically informed antibiotic use.

Parallel to antibiotic exposure, recent evidence also highlights the role of PPIs in facilitating colonization by multidrug-resistant organisms, including CRE [28-30]. PPIs are among the most widely prescribed medications globally, yet up to 70% of their use is considered inappropriate—often prescribed without clear indications or unnecessarily prolonged [31]. In South Korea, the PPI market reached 770 billion KRW in 2022, with studies indicating significant overuse in clinical practice [32-34]. By raising gastrointestinal pH, PPIs reduce natural acid-mediated defenses, promote small intestinal bacterial overgrowth, and alter microbial community structure [11, 35, 36]. These changes are associated with decreased microbial diversity and conditions favoring colonization by antimicrobial-resistant organisms, including CRE [11, 36, 37]. While other acid-suppressive agents such as H2-receptor antagonists may also disrupt gut microbiota, PPIs appear to exert a more pronounced effect [38].

**Fig. 2:**
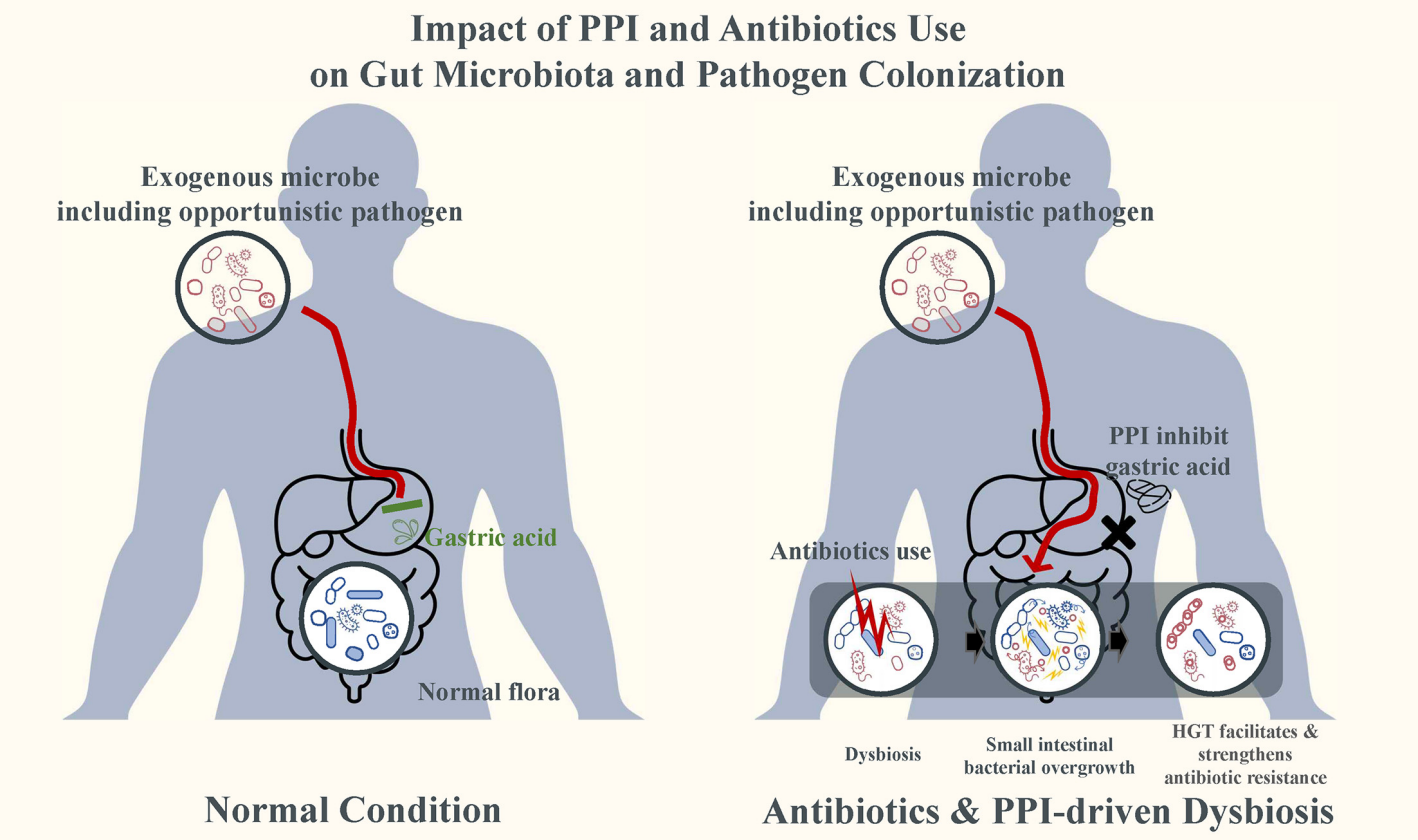
Dual mechanisms by which proton pump inhibitor (PPI) and antibiotic exposure promote dysbiosis, pathogen colonization, and antimicrobial resistance. Under normal conditions (left), gastric acid limits exogenous microbes and balanced gut microbiota provides colonization resistance. In dysbiosis (right), caused by PPIs and antibiotics, two mechanisms promote antimicrobial resistance: (i) Small intestinal bacterial overgrowth (SIBO) enhances proliferation and horizontal gene transfer (HGT) among endogenous pathobionts; (ii) Reduced barriers allow exogenous microbes to colonize and contribute to HGT and antimicrobial resistance genes (ARG) amplification. These processes support the expansion and persistence of multidrug-resistant organisms such as carbapenem-resistant Enterobacteriaceae (CRE).

In a previous metagenomic study conducted by the authors, intestinal colonization with carbapenemase-producing CRE (CP-CRE) was significantly associated with concomitant use of broad-spectrum antibiotics and PPIs [21]. Notably, whole metagenome sequencing revealed that patients receiving both agents exhibited a higher frequency of horizontal gene transfer within the gut microbiota. This suggests that PPI use may accelerate the dissemination of resistance genes under antibiotic pressure. These interactions are illustrated in Fig. 2, which depicts how concomitant exposure to antibiotics and PPIs contributes to CRE colonization and the mobilization of resistance genes. This figure underscores a clinically relevant scenario in which non-antibiotic medications—previously considered microbiologically neutral—can synergize with antibiotics to exacerbate microbiome disruption. In hospitalized or critically ill patients who are frequently exposed to both drug classes, this compounded ecological effect may compromise colonization resistance, promote horizontal gene transfer, and facilitate CRE colonization.

Microbiome-informed stewardship strategies—defined as the integration of gut microbiome status into antibiotic and non-antibiotic prescribing decisions—leveraging MDIs and metagenomic insights, may provide a novel approach to mitigate the emergence and spread of CRE. Tailoring antibiotic and PPI use based on microbiome status has the potential to preserve microbial ecology, limit horizontal gene transfer, and prevent CRE colonization in vulnerable patients.

## Gut Microbiome-Based Strategies for the Development of CRE-Targeted Therapeutics

While the development of novel antimicrobials—such as ceftazidime/avibactam and cefiderocol—remains critical in addressing CRE, their clinical impact is limited by the rapid emergence of resistance [39-41]. As a result, attention has increasingly shifted toward microbiome-based therapeutic approaches that aim to restore or enhance colonization resistance in the gut, thereby preventing CRE acquisition, reducing intestinal carriage, and limiting subsequent infection and transmission. This section highlights mechanistic insights into colonization resistance and, building on these foundations, explores gut microbiome-based strategies for developing CRE-targeted therapeutics, including the use of FMT and the design of defined symbiotic microbial consortia for CRE decolonization.

### Mechanistic Insights into Colonization Resistance against CRE

Colonization resistance against CRE is mediated through both direct and indirect mechanisms [42-45]. Direct mechanisms include nutrient competition, niche exclusion, and the production of inhibitory metabolites such as short-chain fatty acids (SCFAs), which suppress the growth of Enterobacteriaceae. Indirect mechanisms involve microbiota-driven modulation of host immunity, reinforcement of epithelial barrier integrity, and reduction of oxygen availability in the gut environment. A comprehensive understanding of these mechanisms is essential for the rational development of microbiome-based interventions aimed at preventing or eliminating CRE colonization.

Antibiotic-naive microbiota can suppress the expansion of antibiotic-resistant Enterobacteriaceae-including CRE-through acidification of the intestinal lumen and SCFA-mediated intracellular acidification [44, 46]. Elevated levels of SCFAs—such as acetate, propionate, and butyrate—impair CRE fitness by inhibiting both aerobic and anaerobic respiration, which are essential for Enterobacteriaceae proliferation. The combined effects of increased SCFA concentrations and a lowered pH in the proximal colon create an inhospitable environment for CRE expansion. Notably, loss of SCFA production has been linked to the overgrowth of *E. coli* and subsequent bloodstream infection in a patient undergoing allogeneic hematopoietic cell transplantation [46]. Although further studies are needed, restoring SCFA-producing commensals may offer a supportive strategy for limiting intestinal CRE colonization [47].

In addition, several studies have reported that nutrient competition plays a major role in resisting colonization by Enterobacteriaceae, including CRE. Commensal bacteria restrict access to key carbohydrates such as β-glucosidic sugars, glucose, and gluconate—resources exploited by CRE for expansion [48-51]. By reintroducing microbial taxa that occupy similar metabolic niches, colonization resistance may be re-established.

Finally, immune-mediated colonization resistance has been observed in murine models, where a commensal colonization factor (CCF) derived from Bacteroidota promote IL-36γ production by macrophages, enhancing clearance of *K. pneumoniae*, including carbapenem-resistant strains [52]. These findings support the potential role of immune-modulatory microbes in microbiome-based therapeutic strategies for CRE decolonization.

To further illustrate these mechanisms, Table 2 summarizes key studies describing specific commensal strains implicated in colonization resistance against CRE, along with their reported modes of action and experimental evidence.

### FMT for CRE Decolonization

FMT has emerged as a promising therapeutic strategy to restore gut microbial homeostasis and suppress intestinal colonization by CRE, thereby reducing the duration of colonization or limiting clonal expansion in recipients [53, 54]. Clinical studies have reported varying but generally favorable decolonization rates in CRE carriers, with a recent systematic review showing eradication in over 60% of patients at one month post-FMT and nearly 80% by 6 to 12 months [54].

**Fig. 3:**
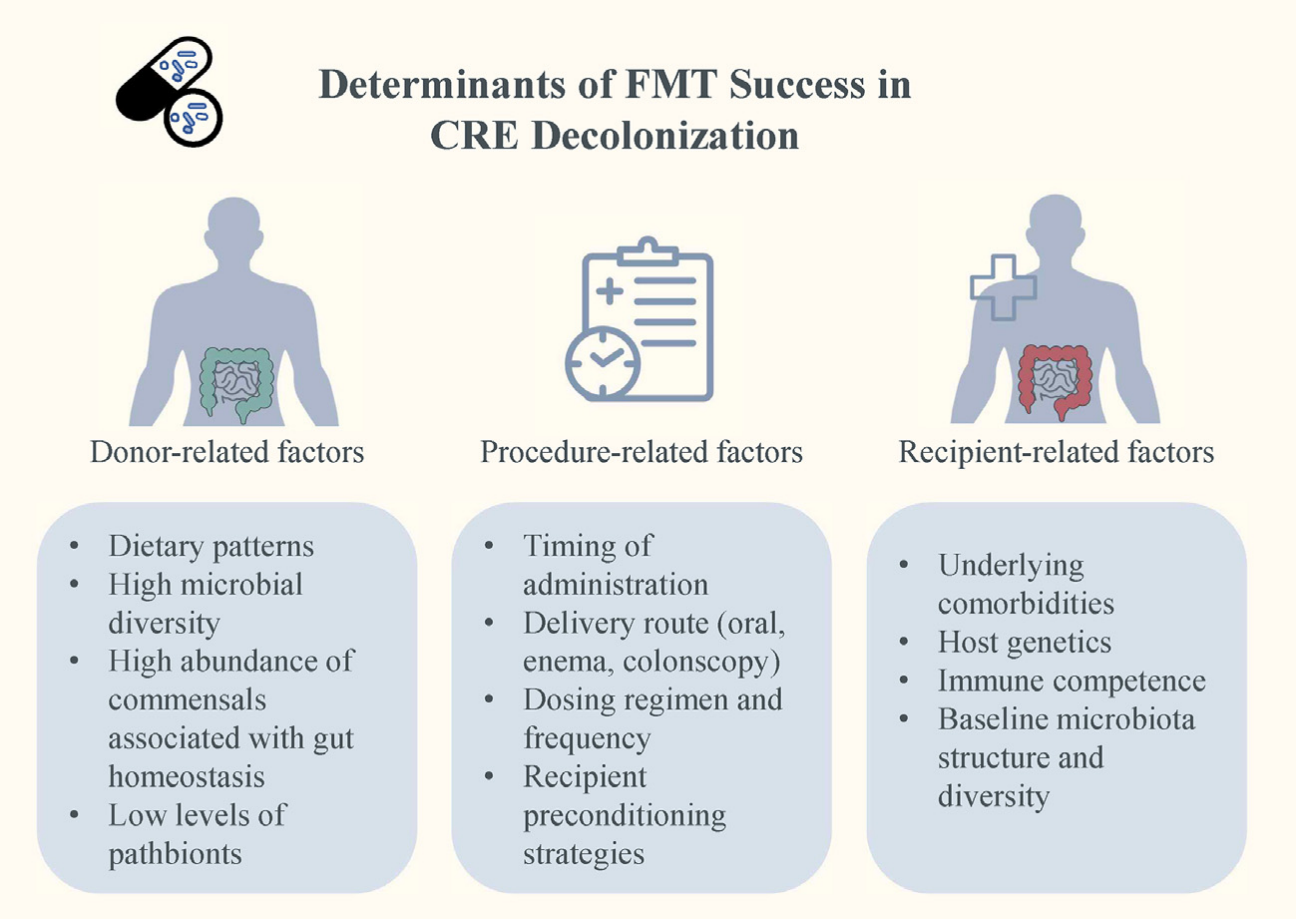
Multidimensional determinants of fecal microbiota transplantation (FMT) success in CRE decolonization. FMT efficacy in carbapenem-resistant Enterobacteriaceae (CRE) decolonization is shaped by donor-, procedure-, and recipient-related factors: donor diet, microbiota diversity, commensal abundance, and low pathobionts; timing, delivery route, dosing, and preconditioning; and recipient comorbidities, immune status, genetics, and baseline microbiota structure. These factors collectively influence engraftment and therapeutic success.

By transferring stool from rigorously screened healthy donors, FMT facilitates the engraftment of beneficial microbial taxa and re-establishment of colonization resistance. The effectiveness of FMT in CRE decolonization is influenced by multiple factors, which can be broadly categorized into donor-, recipient-, and procedure-related variables (Fig. 3) [55]. Donor-related factors include diet, overall microbiota richness, high abundance of beneficial commensals, and low abundance of pathobionts. Recipient-related factors involve exposure to antibiotics and non-antibiotic drugs before and after FMT, underlying diseases, host genetic characteristics, immune status, and baseline microbiota composition and diversity. Procedure-related factors include the timing of FMT, route of administration, dosing frequency, and the use of preconditioning regimens.

Although several studies have evaluated gut microbiota before and after FMT in CRE carriers, few have comprehensively assessed concurrent changes in microbial composition, antimicrobial resistance genes, and metabolic pathways [56-60]. Moreover, most mechanistic investigations of colonization resistance in the context of CRE decolonization have been conducted in animal models rather than in human subjects. In shotgun metagenomic sequencing studies of human gut microbiota pre- and post-FMT, responders typically demonstrate increased taxonomic similarity to donor microbiota, recovery of microbial diversity and metabolic capacity, and a reduction in resistome burden [57, 60].

Despite encouraging results, the routine clinical use of FMT for CRE decolonization remains limited by regulatory restrictions, safety concerns, and challenges in donor screening [54]. In the United States, CDC and the Food and Drug Administration (FDA) regulate FMT under an investigational new drug (IND) framework, with enforcement discretion primarily limited to recurrent *Clostridioides difficile* infections (CDIs) not responsive to standard therapies [61, 62]. In Europe, the absence of harmonized guidance from the European Medicines Agency (EMA) has resulted in considerable variability in regulatory approaches among member states, and discussions are ongoing regarding the potential classification of FMT under the Advanced Therapy Medicinal Products (ATMP) framework [63]. At the global level, WHO has recognized the need for standardized protocols and traceability requirements for substances of human origin, including FMT, although no unified international regulatory framework is currently established [64].

To improve the success rate and predictability of FMT, future efforts should focus on optimizing donor–recipient matching algorithms, developing effective preconditioning protocols, and guiding repeated FMT administration based on real-time microbiome analysis [55]. In addition, the integration of multi-omics approaches and high-throughput culturomics will help elucidate the mechanistic underpinnings of colonization resistance and identify key microbial taxa responsible for successful CRE decolonization [51, 65, 66]. These insights may establish a robust scientific foundation for FMT and support the development of next-generation probiotics, such as defined symbiotic microbial consortia specifically tailored for CRE decolonization.

### Development of Defined Symbiotic Microbial Consortia for CRE Decolonization

Defined microbial consortia— also referred to as next-generation probiotics—are engineered combinations of specific microbial strains that confer colonization resistance [67]. Unlike FMT, these consortia offer standardized composition, quality control, and scalability, making them more suitable for regulatory approval and clinical deployment.

**Fig. 4:**
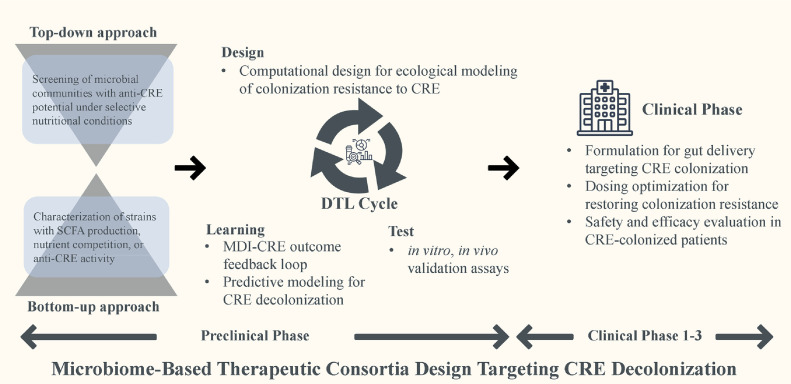
Design framework for developing microbiome-based therapeutic consortia targeting CRE decolonization. Development of microbiome-based therapeutics for carbapenem-resistant Enterobacteriaceae (CRE) follows two complementary approaches: (i) a top-down screening of microbial communities with anti-CRE activity under defined nutritional conditions, and (ii) a bottom-up characterization of individual strains exhibiting relevant functions such as short-chain fatty acid (SCFA) production or nutrient competition. These efforts converge within an iterative Design–Test–Learn (DTL) cycle comprising computational modeling, *in vitro* and *in vivo* validation, and MDI-guided predictive refinement. The workflow is contextualized along a translational continuum, with phase annotations (Preclinical Phase and Clinical Phases 1–3) added to clarify the research-to-clinic progression.

Development strategies follow two main paradigms (Fig. 4): (i) a top-down, which selects high-performing microbial communities from complex microbiota based on functional screening, and (ii) a bottom-up, which assembles individually characterized microbial strains with complementary metabolic and immunomodulatory functions [67, 68]. Both approaches utilize iterative “Design-Test-Learn” cycles that incorporate computational modeling, along with *in vitro* and *in vivo* validation steps [67, 69]. Once optimized consortia are finalized during the preclinical phase, the clinical phase focuses on determining key formulation parameters—including preparation methods, dosing strategies, and delivery routes—followed by systematic evaluation in clinical trials [67]. Fig. 4 outlines this translational continuum, emphasizing the importance of rational design, functional validation, and regulatory readiness in accelerating the clinical implementation of microbiome-based therapeutics for CRE decolonization.

Rebyota and Vowst have received FDA approval, and notably, VE303—a defined symbiotic microbial consortium consisting of eight commensal *Clostridia* strains—is currently undergoing phase 3 clinical trials to evaluate its efficacy and safety in preventing recurrent CDI [70, 71]. Building on these advances, symbiotic microbial consortia are being explored for the targeted decolonization of Enterobacteriaceae, including CRE. In a recent study, Furuichi *et al*. developed a rationally assembled 18-strain commensal bacterial consortium derived from healthy human donors that effectively suppressed intestinal colonization by *K. pneumoniae* and *E. coli*—representative Enterobacteriaceae species frequently associated with CRE [51]. The consortium exerted its effects by restoring colonization resistance through ecological control, specifically by limiting the availability of gluconate, a key nutrient utilized by Enterobacteriaceae for expansion. In murine models, this approach not only reduced intestinal CRE burden but also mitigated inflammation associated with gut dysbiosis. These findings underscore the promise of defined symbiotic microbial consortia as microbiome-based therapeutics for CRE decolonization and infection prevention.

From a regulatory perspective, defined microbial consortia and other live biotherapeutic products are subject to increasingly structured evaluation frameworks. In the United States, the FDA classifies such products as biological products and requires submission under an IND application, with recent approvals granted for microbiota-based therapies such as Rebyota and Vowst [72, 73]. In Europe, the EMA has not yet established harmonized guidance specific to microbial therapeutics, but defined consortia may be regulated either as ATMP or as biological medicinal products, depending on their composition and intended use [63]. Ongoing discussions within the EMA Innovation Task Force aim to clarify regulatory pathways and facilitate the development of safe and effective microbiome-based interventions [63].

## Conclusion

CRE remain a critical public health threat due to their resistance to last-line antibiotics, capacity for prolonged gastrointestinal colonization, and high transmissibility in healthcare settings. As antibiotic development struggles to keep pace with resistance, microbiome-based strategies have emerged as promising adjuncts to conventional approaches for CRE control. Disruption of the gut microbiota—often driven by antibiotics and non-antibiotic drugs like PPIs—compromises colonization resistance and facilitates CRE acquisition and persistence. MDIs offer a novel tool for identifying patients at risk of CRE colonization or infection, guiding targeted surveillance and stewardship interventions. Incorporating MDIs into clinical practice may enable precision prevention strategies, particularly in high-risk populations such as ICU patients. Therapeutically, FMT has demonstrated efficacy in eradicating CRE in select cases, although broader clinical implementation remains limited by safety, regulation, and standardization barriers. More recently, defined symbiotic microbial consortia—composed of rationally selected commensal strains—have shown potential in preclinical models to reduce CRE colonization by restoring ecological balance and outcompeting pathogens.

As the field advances, future priorities include validating MDIs in prospective clinical trials, optimizing donor selection and procedural protocols for FMT, and accelerating the development of scalable, regulatory-compliant microbial consortia. Integration of metagenomics, metabolomics, and culturomics will be essential for identifying mechanistic drivers of colonization resistance and refining therapeutic targets. In the face of rising multidrug resistance, gut microbiome-based approaches represent a promising frontier for CRE risk prediction, prevention, and decolonization—offering new tools to combat one of the most urgent threats in the post-antibiotic era.

In summary, this review offers several new perspectives to advance the field of CRE control. First, it emphasizes the clinical potential of MDIs not only as predictive biomarkers of ecological vulnerability but also as tools to guide real-time infection prevention and antimicrobial stewardship decisions. Second, it highlights the underrecognized impact of non-antibiotic medications, particularly proton pump inhibitors, in driving microbiome disruption and facilitating CRE colonization—suggesting a need for more integrated stewardship frameworks. Third, it underscores the translational promise of defined symbiotic microbial consortia as scalable, standardized alternatives to fecal microbiota transplantation for restoring colonization resistance. Collectively, these insights contribute to a more holistic, microbiome-informed strategy for preventing and decolonizing CRE in the post-antibiotic era.

## Figures and Tables

**Table 1:** Comparative summary of studies relevant to the Microbiome Disruption Index (MDI).

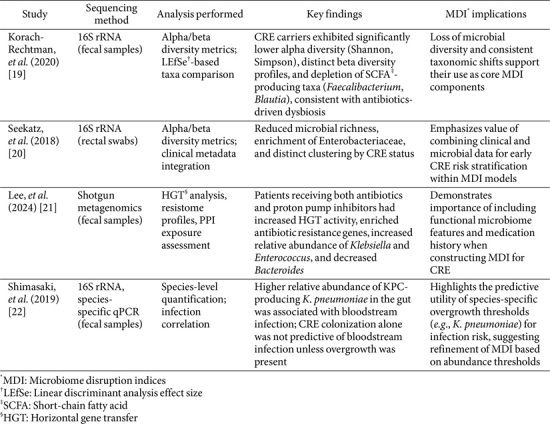

**Table 2:** Summary of key mechanistic studies on microbiota-mediated colonization resistance to CRE and other multidrug-resistant Enterobacteriaceae.