# Corrigendum to: Gut Microbiome-Based Strategies for the Control of Carbapenem-Resistant Enterobacteriaceae

**DOI:** 10.4014/jmb.2025.3511.C01

**Published:** 2025-11-04

**Authors:** Imchang Lee, Bong-Soo Kim, Ki Tae Suk, Seung Soon Lee

**Affiliations:** 1 Division of Infectious Diseases, Department of Internal Medicine, Hallym University Chuncheon Sacred Heart Hospital, Hallym University College of Medicine, Chuncheon, Republic of Korea; 2 Department of Nutritional Science and Food Management, Ewha Womans University, Seoul, Republic of Korea; 3 Division of Gastroenterology and Hepatology, Department of Internal Medicine, Hallym University Chuncheon Sacred Heart Hospital, Hallym University College of Medicine, Chuncheon, Republic of Korea; 4 Institute for Liver and Digestive Diseases, Hallym University, Chuncheon, Republic of Korea

J. Microbiol. Biotechnol. 2025. 35: e2406017

https://doi.org/10.4014/jmb.2406.06017

The authors regret that the following grant information was inadvertently omitted from the Acknowledgements section of the article. The correct acknowledgement should have read:

**This research was supported by the Hallym University Research Fund and the Korea National Institute of Infectious Diseases, Korea National Institute of Health [2023-ER2107-02].**

The authors would like to apologies for any inconvenience caused. (http://jmb.or.kr)

