# Corrigendum to: Gut Microbiome-Based Strategies for the Control of Carbapenem-Resistant Enterobacteriaceae

**DOI:** 10.4014/jmb.2025.3512.C01

**Published:** 2025-12-09

**Authors:** Imchang Lee, Bong-Soo Kim, Ki Tae Suk, Seung Soon Lee

**Affiliations:** 1 Division of Infectious Diseases, Department of Internal Medicine, Hallym University Chuncheon Sacred Heart Hospital, Hallym University College of Medicine, Chuncheon, Republic of Korea; 2 Department of Nutritional Science and Food Management, Ewha Womans University, Seoul, Republic of Korea; 3 Division of Gastroenterology and Hepatology, Department of Internal Medicine, Hallym University Chuncheon Sacred Heart Hospital, Hallym University College of Medicine, Chuncheon, Republic of Korea; 4 Institute for Liver and Digestive Diseases, Hallym University, Chuncheon, Republic of Korea

The authors regret that the following grant information was inadvertently omitted from the Acknowledgements section of the article. The correct acknowledgement should have read:

**This research was supported by the Hallym University Research Fund and the Korea National Institute of Infectious Diseases, Korea National Institute of Health [2023-ER2107-02 and 2024-ER2208-01].**

The authors would like to apologies for any inconvenience caused. (http://jmb.or.kr)

